# Parallel/Opposed Editorial: DMP/residency programs are more sustainable than MPAs for the future of the medical physics profession

**DOI:** 10.1002/acm2.12439

**Published:** 2018-08-17

**Authors:** Chengyu Shi, Brent C. Parker, Yi Rong

**Affiliations:** ^1^ Department of Medical Physics Memorial Sloan Kettering Cancer Center Basking Ridge NJ USA; ^2^ Division of Physics and Engineering Department of Radiation Oncology The University of Texas Medical Branch Galveston TX USA; ^3^ Department of Radiation Oncology University of California Davis Comprehensive Cancer Center Sacramento CA USA

## 1 INTRODUCTION

Medical Physics has been a popular profession to enter for science program graduates, which include students from medical physics, physics, biomedical engineering, chemistry, etc. For many years, there were minimal academic limitations for students that graduated from a nonmedical physics program to get into this profession. However, things have changed since the American Board of Radiology (ABR) created strict eligibility requirements for taking the certification examinations, with the completion of a CAMPEP (Commission on Accreditation of Medical Physics Education Programs) accredited graduate program beginning in 2012 and a CAMPEP accredited residency program beginning in 2014. Meanwhile, the first Professional Doctorate in Medical Physics (PDMP or DMP for simplicity) was established and admitted their first student in the Fall of 2009. The same program received CAMPEP accreditation in the following year. Also, around the same time, the concept of Medical Physicist Assistants (MPA) was introduced into our field first in the diagnostic subfield and then legitimized in the Medical Physics profession in 2013 through the formation of Medical Physics Practice Guideline (MPPG)‐3. Residency continues to be a mainstream option for most eligible graduates, yet the existence of DMP and MPA has brought heated debates in the past years. As it is approaching a 10‐yr mark since the inception of DMP/MPA, a decline in DMP positions and an increase in MPAs have been observed in our field. This begs the question: “whether DMP/residency or MPA is sustainable for the future of the medical physics profession?” Herein, we have invited Dr. Chengyu Shi arguing for the proposition that “DMP/residency programs are more sustainable than MPAs” while Dr. Brent Parker argues against it.

Dr. Chengyu Shi is an associate attending medical physicist, and the lead physicist overseeing clinical physics operations at Memorial Sloan Kettering's outpatient locations in Basking Ridge and Monmouth, New Jersey. His research interests are in Monte Carlo simulation, virtual human phantom development and applications, special treatment techniques including stereotactic body radiotherapy, stereotactic radiosurgery, and more. Dr. Shi received his Ph.D. (2004) in nuclear engineering and science from Rensselaer Polytechnic Institute in Troy, New York, and finished his residency training at the University of Arkansas for Medical Sciences. He taught and mentored residents at the University of Health Science Center at San Antonio, Texas, for several years.

Dr. Brent Parker obtained his Ph.D. (2004) and M.S. (2001) in Medical Physics from the University of Texas Health Science Center at Houston and M.D. Anderson Cancer Center Graduate School of Biomedical Sciences and his B.S. (1997) in Physics from Louisiana Tech University. He has been involved in both clinical and academic medical physics in his entire career. Although arguing against the presented statement, he is a proponent of medical physics residency programs. He helped establish the Medical Physics Residency Program at Mary Bird Perkins Cancer Center in Baton Rouge, LA and served as its Program Director. He is currently an Associate Professor and the Director of Physics in the Department of Radiation Oncology at the University of Texas Medical Branch in Galveston, TX. Dr. Parker has served the AAPM at both the national and chapter levels in a variety of committee and elected positions. He is certified by the American Board of Radiology in Therapeutic Radiologic Physics.

## 2 OPENING STATEMENT

### 2.A Chengyu Shi, Ph.D

The American Association of Physicists in Medicine (AAPM)^1^ defines “medical physicist” as “an individual who is competent to independently provide clinical professional services in one or more of the subfields of medical physics.” In addition, AAPM poses two minimum requirements toward credentialing a qualified medical physicist (QMP), a master or doctoral degree, and a national certification, that is, the ABR. Starting the year of 2012, the ABR mandated a 2‐yr residency training, in addition to a degree completion from a CAMPEP‐accredited program, for receiving the certification as a QMP.^2^ Such stringent requirements resulted from an overall understanding in the field that a QMP carries such a critical responsibility toward patient safety in clinic, and any inadequate training may result in detrimental events. On a contrary, a new job position has emerged in recent years and is given a title “medical physicist assistant (MPA)”. A MPA is defined as “an individual who works under the supervision and responsibility of a QMP and is not currently on a path to become a board‐certified medical physicist”.^3^ The need of MPAs resulted from the imbalanced supply of eligible medical physicists graduating from residency programs. The number of resident positions is far less than the increased demand of clinical medical physicists.^4^ At the same time, however, the increasing number of graduating students almost doubles the number of residency positions, which ultimately leaves a large pool of unmatched students. Therefore, it sounds reasonable that the unmatched MP students, and other potential candidates who are not qualified for a QMP, can have a temporary position during their transition, and the MPA is perfect for balancing this need from both ends. However, from a clinical safety point of view, it defeats the high standards that have been posed on the medical physics profession for our goal of patient safety. One may argue that MPA is only to work under the supervision of a QMP. But who is there to monitor and what guidelines are available for clearly defining the word “supervision”? It has been very clear to us that only a graduate from doctorate degree in medical physics (DMP) or a resident program is eligible for the ABR board certification. That entails at least 5 yr of training in medical physics subfields. However, MPA may serve as a shortcut and ultimately hurt the entire profession. Therefore, I herein argue that MPA is not sustainable in our medical physics profession. The reasons are multifold.

First, medical physics is an ever‐evolving field. The knowledge in this field is expanding exponentially and the emerging new technology is fast‐replacing old ones. Looking back 10 yr, we are leaping from three‐dimensional (3D) era to IMRT (intensity‐modulated radiation therapy) and IGRT (image‐guided radiation therapy) era. Looking forward, we may advance to an era with multimodality (such as MRI‐linac, MRI‐PET, etc.) and artificial intelligence (AI). Medical physicists are the core in the success of these advances, which require a medical physicist to be able to fast absorb new knowledge and even lead the development of those technologies. It may be challenging for MPA with limited relevant knowledge and clinical training. DMP/resident trainees have been equipped with dedicated training and knowledge for adapting to the versatile environment in medical physics field and also made themselves ready for the future of medical physics research and development. In addition, medical physicists play an important role in new technology development. For example, the superposition/convolution method was developed by Rockwell Mackie,^5^ who started his career as a clinical medical physicist in the field. Rotational intensity‐modulated fan‐beam delivery, that is, TomoTherapy, and rotational intensity‐modulated cone‐beam delivery, that is, volumetric‐modulated arc therapy (VMAT), developed between 1993 and 1995,^6^, ^7^ was both invented by medical physicists with profound understanding in the field and clinical training. The medical physics profession might become obsolete and easily replaceable if no medical physics pioneers broaden new horizons.

Second, a QMP is needed to run routine clinical duty, while a MPA does not meet the requirement. When the system becomes more complex, the higher standard and requirements will need further clinical training and qualified individuals to confirm that the clinical work is done correctly. Some clinical workload may be shared by an MPA, but it is still QMP's responsibility to double check and sign off. It may not save time for QMP and it may introduce extra risk of making errors if the work was done by an insufficiently trained individual. In addition, beyond the limited tasks that can be completed under the supervision of a QMP, an MPA may have limited ability or motivation to put into field innovation, which may lead to low job satisfaction and fast turnaround. Ultimately, it may take more time for a QMP to train and supervise a fresh MPA instead of saving time on routine clinical work. One may argue that DMP and resident are also temporary positions, yet they have a clear career path to becoming a QMP and the training they received is all substantially counted toward their career development. The career path for them is far more promising. For example, they can obtain ABR board certification, become a clinical physicist, conduct research, and even become a manager or leader in the field. The career path will motivate DMP and residents to improve themselves and provide better work quality to ready them for their next step. The entire medical physics profession has been nurtured and growing with such motivation, while the shortcut to MPA may hurt or kill it.

In conclusion, based on the above‐listed reasons, the DMP/resident program may be more sustainable for the future of the medical physics profession. MPAs may provide important support in the clinic for some centers with staff shortages, but it is a temporary solution and may not be positively serving the medical physics profession in a long run.

### 2.B Brent C. Parker, Ph.D

The 2014 ABR mandate for completion of an accredited medical physics residency program for board certification created a bottleneck in the path for entry into the field of medical physics. From 2012 to 2016, there was an average of 270 graduates of medical physics MS/MSc and Ph.D. graduate programs per year.^8^ The most recent data from the national residency matching program shows that in 2018, 272 applicants registered for the match while 204 applicants ultimately participated. Of these, 116 applicants matched to the 129 offered positions.^9^ Since 2015, approximately 110 positions were filled each year, resulting in an average match rate of 52% from 2016 to 2018.^10^ Therefore, residency programs are accommodating roughly half the graduates of medical physics graduate programs. There is some uncertainty in the numbers due to contributions from DMP programs, but that impact is minimal with only five accredited programs.^11^ Unfortunately, the data are not subdivided into therapy and imaging, but it still provides a view of the profession's future workforce supply.

Using radiation oncology physics as an example, workforce analyses project that the profession will need approximately 180 new radiation oncology medical physicists for the period 2020–2030.^12^, ^13^ CAMPEP and match program data show that the profession is not matching a total number of residents annually (therapy and imaging combined) to meet workforce needs with 99 accredited therapy programs and 24 accredited imaging programs. From 2013 to 2017, residency programs increased annually at a rate of 6.8 for therapy and 3 for imaging.^14^ It is uncertain whether this rate of growth can be maintained long term or whether existing programs will continue to operate at their current enrollment levels. The situation may even be worse on the imaging side where there are currently not enough residency positions available through academic institutions. This puts an emphasis on private imaging groups generating additional positions. A potential conflict emerges where consulting groups are training physicists who will compete with them for contracts, potentially in the same geographic region.

Unless there is a significant growth in the number of accredited residency programs, we cannot meet future workforce needs. However, a limiting factor of program growth is the cost of program operation. Data indicate that each therapy resident provides an average of 0.375 clinical medical physicist FTE.^15^ That program's model also indicated that approximately 3–4 residents could be supported for the cost of one board‐certified clinical medical physicist. This makes the cost per FTE clinical physics support provided by residents approximately equal to that of a staff physicist. What is not included in this analysis, however, is the staff percent FTE allocated to program training and administration. This makes the residency program at best cost neutral, but more likely a net cost to the sponsoring institution.

One potential solution to the cost issue was the creation of DMP programs, where the cost of program operation is transferred to the student in the form of tuition. While a viable option in theory, this solution has not materialized. One potential obstacle for creation of new DMP programs is that they may require review and approval from department, university, and state entities as new degree programs.

A more practical approach is the use of MPAs to provide clinical workload support. While the MPA title has more recently come into standard use, the role has been around for decades, going by titles such as “QA dosimetrist”. In reality, MPAs provide a better long‐term return on investment than residents. Residency programs require training for new residents every year, and after 2 yrs, the residents have left the program. There is no long‐term continuity and service from the trained individuals who move into the job market. Ideally, once a MPA is trained in clinic procedures, they will provide longer service to the institution than the 2‐yr residency cycle. Using the staffing example above, for the cost of a board‐certified clinical medical physicist, an institution can hire 2–3 MPAs. Assuming that a fully trained MPA (at least 1 yr experience) provides the same clinical support as a second‐year resident, this provides 1.0–1.5 clinical FTEs. In this scenario, the MPA model provides more clinical support than a residency program per unit of cost. Combined with the potentially reduced turnover compared to residents, MPAs make a more appealing long‐term solution.

The ABR bottleneck leaves a large pool of medical physics graduates who do not have job opportunities or pathways into the profession. Unfortunately, as long as there are an inadequate number of residency positions, these graduates will need job opportunities. The MPA role offers those opportunities. AAPM already recognizes the impact that MPAs have on the profession and is addressing it with items like Medical Physics Practice Guideline #7 and Professional Policy 29‐A. It is no longer a question of whether MPAs have a role in the future of medical physics, but whether the profession can move forward without them.

## 3 REBUTTAL

### 3.A Chengyu Shi, Ph.D

I agree with Dr. Parker's statement that there is a bottleneck and shortage of DMP/residency program for the coming graduated students. However, MPA may serve as a temporary solution for this bottleneck situation, but whether it is a sustainable way for the future of medical physics profession is arguable. I agree with my opponent that the DMP program may be slow to buildup and takes long time to meet the needs. However, the residency program can be expanded and grow faster. There are several other approaches that the medical physics field can implement to solve the shortage issue and provide sustainable ways for the future of medical physics profession.

First of all, CAMPEP can continue to approve more residency programs. Current residency programs are mainly developed by academic centers. However, stand‐alone clinics or private centers also have QMPs who can mentor residents. Those centers should be able to start a residency program or affiliate with the existing residency programs to provide more training positions for the graduates. The private centers have not seen the benefits of residency program right now possibly due to the limited budget of the department and the overwhelmed workload of medical physicists. They usually have tighter budget and their staffing model is not following ASTRO recommendations.^16^ Yet, the overwhelmed workload may be a driving force for those medical physicists to educate their managers and physicians to start residency programs for economic and safety considerations. However, if MPA is an option, chances are the managers might very well go with hiring MPAs instead of opening residency programs.

Secondly, the existing DMP/residency program can provide more positions. The current CAMPEP programs only approve certain number of residency positions, which are usually lower than the maximum positions a center can afford. CAMPEP can survey the existing programs and allow the programs to expand their residency positions. Some large academic centers have satellite centers, which can provide equivalent training. Moreover, some private centers are now either affiliating with or joining larger centers (MD Anderson makes a perfect example). Those affiliated centers should have the ability and resources to provide residency training under the umbrella of the larger center.

The medical physics society should have a structure for training our future medical physicists. For research and development, post doc and DMP are necessary and should be encouraged. Residency training is also necessary to gain clinical service experience. MPAs are usually recruited due to the staff shortage and/or high workload. However, they may stay in this position and perform routine work, or with sufficient training, be redirected to other medical physics‐related domains, such as dosimetrists for treatment planning or IT staff for system maintenance. As mentioned previously, even well‐trained MPAs have the ability for routine clinical work, but may still have limited in‐depth knowledge or may not be eligible in taking the ABR board. Allowing MPA positions in our profession seems to be an economic and easy solution in a short term, but it may hurt the future growth of DMP/residency programs. The need and shortage of QMP are the fundamental drive force for the growth of our DMP/residency programs. However, once these needs are met with the short‐term solution of MPA, who will have the motivation to keep promoting DMP/residency programs? In summary, the MPAs can provide certain service to the clinic but is not a sustainable solution for medical physics field in a long run.

### 3.B Brent C. Parker, Ph.D

I completely agree with my colleague that our profession is dynamic and we must maintain the high standards we have established for patient care. That is precisely why we need QMPs to ensure safe and effective patient care with current and emerging technologies. However, medical physicists typically operate as part of a larger team, department, and institution. We live in a time of increased fiscal responsibility and one of our primary professional goals is to be good stewards of not only the profession but also of our institutional resources. We can achieve that by providing the highest quality care for our patients in a cost‐effective manner. Many of the technological leaps that my colleague identifies (IMRT, IGRT, etc.) required a significant QMP involvement for clinical development and implementation. However, as these technologies matured, much of the operational effort has become routine QA and measurement. Turning these tasks over to well‐trained and supervised MPAs will free up valuable and limited resources, measured in both QMP expertise and financial considerations, to manage the care of our current patients while also focusing on the next generation of technological advancement and implementation.

I do not argue that MPAs can, or should, replace DMPs/residents as they represent the pool of QMPs who will lead our profession into the future. It would be great to live in a world where all of the clinical work are performed by QMPs with the training, continuing education, and ongoing evaluation that goes along with those credentials. Unfortunately, the ABR requirement for completion of an accredited residency program has put us in a bind. We need to be honest with ourselves and acknowledge that the residency programs simply do not have the capacity to meet our future staffing needs. I sincerely hope that changes, but in the interim, we cannot assume that it will. Without the use of MPAs, there is a real risk of QMPs becoming overworked to provide clinical service, residency training, and technology advancement. This is exactly the type of scenario that leads to increases in error rates and decreased job satisfaction.^17^, ^18^ I think we all can agree that it is a disservice to our profession and, more importantly, to our patients.

AAPM Medical Physics Practice Guidelines (MPPG's) #7 and #10 will soon be published and address this issue from different directions, and there is a good reason why these documents are expected to be published almost simultaneously. MPPG #7 will focus on the MPAs (supervision, competency, etc.) while MPPG #10 will define the scope of practice for clinical medical physicists. These two documents will clearly establish roles, responsibilities, expectations, etc., for QMPs and MPAs to ensure that the high standard of care our patients expecting from our profession is being met. In reality, if MPA training and evaluation are done thoroughly and correctly, it should look very similar to residency program training plans to establish competency in various clinical procedures. Given that we both argue that a significant source of MPAs will come from the pool of medical physics graduates who could not find residency positions, how can one argue that they would be any less competent or provide a lower standard of care than graduates of residency programs? Even if you make the assumption that the residency programs are taking the best candidates, they are still able to accommodate less than 50% of those completing graduate programs. That leaves a lot of competent people who could ably fill available MPA positions.

I see the role of MPAs as similar to medical “mid‐level providers” such as nurse practitioners (NP's) and physician assistants (PA's). My colleague implies that MPAs do not provide the high standards that our profession has established. Studies have shown that NPs and PAs can provide comparable care to primary care physicians.^19^ With adequate supervision and training on specific tasks, why should we expect MPAs to be any different?

### CONFLICT OF INTEREST

The authors declare no conflicts of interest.
